# Genetic Causes of Qualitative Sperm Defects: A Narrative Review of Clinical Evidence

**DOI:** 10.3390/genes15050600

**Published:** 2024-05-08

**Authors:** Andrea Graziani, Maria Santa Rocca, Cinzia Vinanzi, Giulia Masi, Giuseppe Grande, Luca De Toni, Alberto Ferlin

**Affiliations:** 1 Department of Medicine, University of Padova, 35128 Padova, Italy; andrea.graziani.3@phd.unipd.it (A.G.); giulia.masi@unipd.it (G.M.); luca.detoni@unipd.it (L.D.T.); 2 Unit of Andrology and Reproductive Medicine, University Hospital of Padova, 35128 Padova, Italy; mariasanta.rocca@aopd.veneto.it (M.S.R.); cinzia.vinanzi@aopd.veneto.it (C.V.); giuseppe.grande@aopd.veneto.it (G.G.)

**Keywords:** male factor infertility, idiopathic infertility, sperm motility, sperm morphology, asthenozoospermia, teratozoospermia

## Abstract

Several genes are implicated in spermatogenesis and fertility regulation, and these genes are presently being analysed in clinical practice due to their involvement in male factor infertility (MFI). However, there are still few genetic analyses that are currently recommended for use in clinical practice. In this manuscript, we reviewed the genetic causes of qualitative sperm defects. We distinguished between alterations causing reduced sperm motility (asthenozoospermia) and alterations causing changes in the typical morphology of sperm (teratozoospermia). In detail, the genetic causes of reduced sperm motility may be found in the alteration of genes associated with sperm mitochondrial DNA, mitochondrial proteins, ion transport and channels, and flagellar proteins. On the other hand, the genetic causes of changes in typical sperm morphology are related to conditions with a strong genetic basis, such as macrozoospermia, globozoospermia, and acephalic spermatozoa syndrome. We tried to distinguish alterations approved for routine clinical application from those still unsupported by adequate clinical studies. The most important aspect of the study was related to the correct identification of subjects to be tested and the correct application of genetic tests based on clear clinical data. The correct application of available genetic tests in a scenario where reduced sperm motility and changes in sperm morphology have been observed enables the delivery of a defined diagnosis and plays an important role in clinical decision-making. Finally, clarifying the genetic causes of MFI might, in future, contribute to reducing the proportion of so-called idiopathic MFI, which might indeed be defined as a subtype of MFI whose cause has not yet been revealed.

## 1. Male Factor Infertility and Semen Examination

Infertility, defined as the inability to conceive after 12 months of regular and unprotected intercourse [1], is a well-recognized condition affecting 10–15% of couples worldwide. About 50% of the overall cases involve one or more male factors, either alone or combined with a female factor [2,3]. Male factor infertility (MFI) has a variety of causes, ranging from genetic mutations to lifestyle factors, testicular impairment, systemic diseases, or drugs. In fact, MFI represents a perfect example of a complex disease with a substantial genetic basis, with genetic factors being the cause, either alone or in combination with other factors, of 10–15% of the cases [4,5].

Relatively few genetic analyses are currently recommended in clinical practice. Those that are recommended are represented mainly by factors affecting global spermatogenic activity (such as chromosomal aberrations, Y chromosome microdeletions, and genes causing hypogonadotropic hypogonadism) or sperm transit (such as CFTR mutations in cases of the bilateral absence of the vas deferens) [2,5]. Advances in the understanding of MFI and its genetic components will soon enable the clinical application of genetic tests to unravel the causes of many other forms of reduced fertility in males.

Until then, idiopathic MFI, defined as abnormal semen results without evidence of a clear cause or risk factor, still accounts for about 30–50% of the cases [2,3]. Indeed, although it is pretty evident that the term “idiopathic” should be assigned only after a thoughtful and complete diagnostic process, guidelines do not agree on the correct management of MFI, which is often limited to semen analysis [6]. Furthermore, the relationship between alterations in semen parameters and male fertility potential is not so evident, as emphasised in the last edition of the WHO manual for semen examination and interpretation [7], which also stressed that semen analysis is just the starting point of the diagnostic process for the male partner of an infertile couple. “Normal” semen results do not automatically define a man as “fertile”, and “abnormal” semen results do not define him as “infertile”. 

In this regard, it is worthwhile to highlight that the WHO has not defined “normal values” for semen parameters but has instead provided “reference limits”, with “abnormal” values falling below the fifth percentile. This important concept is often misinterpreted [8]. The reference limits, together with other clinical and diagnostic assessments, are just an indication used to aid in the clinical interpretation (together with the other clinical and diagnostic assessments) of whether a semen parameter is lower than 5% of the results of the reference population or not. Indeed, it is also essential to interpret semen analysis by evaluating different parameters together. Therefore, the fifth percentile reference limit is not a specific cut-off between infertile and fertile men, and results lower than the limits might be compatible with natural fertility, whilst, on the other hand, results above the limits might still be associated with reduced fertility potential [8]. This is also because standard semen analysis analyses rough sperm characteristics, such as the number, motility, and morphology; however, many other functional parameters are involved in male fertility potential. 

For the reasons explained above, the WHO has eliminated terms such as “oligozoospermia”, “asthenozoospermia”, and “teratozoospermia”, which were used to indicate when sperm numbers, the percentage of motile sperm and the percentage of sperm with normal morphology were below the fifth percentile of reference values. Similarly, the term “normozoospermia” should not be used simply because there might be impairments of some parameters that have not been examined in a standard semen analysis [8].

In addition to the complete absence of sperm in the ejaculate (azoospermia), there are other extreme situations in which the reference limits lose their significance in the clinical interpretation of semen analysis and in the evaluation of male fertility potential. These are represented by the presence of sperm with absolute (100%) immotility and the presence of sperm with absolute (100%) malformed morphology, especially when all of the cells present an identical alteration in head, neck/midpiece, or tail morphology (monomorphic forms). In these cases, not only is natural fertility impossible or strongly reduced, but the possibility of a genetic cause is very high, mainly when they are found as isolated alterations in semen analysis.

In the last few years, significant advances have been made in the understanding of the genetic basis of these qualitative sperm defects (sperm with low motility and/or with malformed morphology). However, the potential routine application of genetic tests related to these conditions remains controversial. In this review, we will present the current knowledge on the pathophysiological mechanisms related to sperm motility and sperm morphology and then focus on the genetic basis of qualitative sperm defects, with critical considerations regarding the clinical application of new genetic tests. 

## 2. Sperm Motility and Genetics of Reduced Sperm Motility

### 2.1. Sperm Motility

The sperm cell is morphologically divided into two parts: the head and the flagellum. The structure of the head will be discussed later. The sperm flagellum, which is responsible for sperm motility, is subdivided into four major regions: the connecting piece, the midpiece, the principal piece, and the endpiece [9]. The endpiece of the sperm is only composed of the axoneme, devoid of any peri-axonemal structure, whilst the other parts are surrounded by several structures, such as the mitochondrial sheath, the outer dense fibres, and the fibrous sheath. A comprehensive review of the structure of flagellum is beyond the aim of this paper and might be delved into in a few recent manuscripts [9,10,11]. The motility mechanism of sperm is guaranteed by flagellar beating, which consists of a wave propagating from the base to the tip of the flagellum [12] due to the action of the so-called “motor complex”—made up of the axoneme (with the 9 + 2 structure) [9]. In his context, there are two multiprotein ATPase complexes, dynein arms—outer and inner—which are projections departing from microtubule A, which interact with microtubule B, further allowing sperm motility [10]. Other structures that regulate sperm motility are central pair complex and radial spokes.

The morphological and/or functional defects of the sperm flagellum induce reduced sperm motility. The semen analysis in the men of infertile couples frequently shows reduced sperm motility, both as an isolated finding and in association with other semen alterations, such as a low number of sperm or a high percentage of malformed sperm. In our series of more than 5000 infertile couples, total sperm motility and/or progressive motility below the fifth WHO percentile as isolated semen alteration was found in about 12% of the males [13]. 

Low sperm motility has numerous causes, and the main pathophysiologic mechanisms include those altering mitochondrial function, the flagella, seminal fluid characteristics (such as semen viscosity), the presence of sperm antibodies, and seminal tract infection/inflammation. From a clinical point of view, reduced sperm motility should be distinguished from low sperm vitality, as in this case, the sperm is immotile just because it is dead. Fundamental problems in sperm motility are indeed represented by immotile but viable sperm.

**Figure 1:**
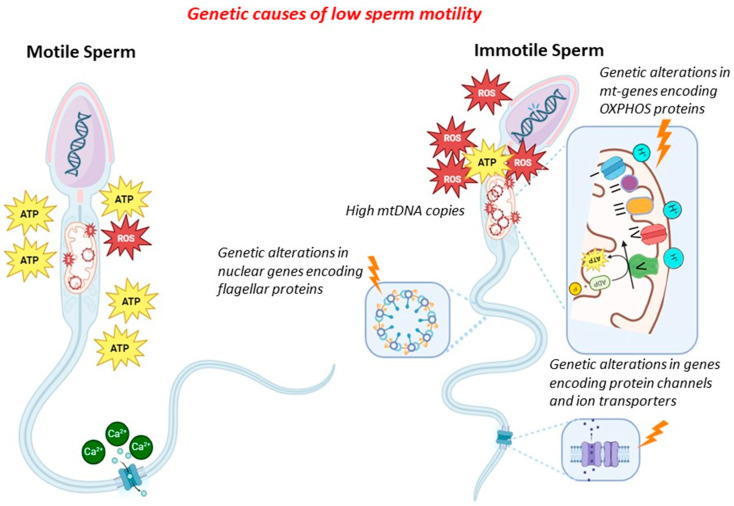
Genetic causes of reduced sperm motility.

In general, genetic factors might contribute to low/absent sperm motility by altering (i) sperm mitochondrial DNA (CNV, mutations and deletions, integrity), (ii) mitochondrial proteins (gene mutations in proteins of the mitochondria), (iii) ion transport and channels (gene mutations in proteins of ion channels), and (iv) flagellar proteins (gene mutations in proteins of the flagella). In the latter cases, sperm immotility is associated with morphological alterations of the flagella (therefore, it combines absent motility with malformed sperm), and this condition is known as multiple morphological alterations of flagella (MMAF). Since many proteins of the sperm flagella are components of the cilia of other cells (in particular, bronchial cells), low/absent sperm motility might also be associated with different clinical conditions (syndromic forms), known as primary ciliary dyskinesia (PCD). The mechanisms exposed above are shown in Figure 1.

### 2.2. Sperm Mitochondrial DNA Content and Proteins

The mitochondrion is a subcellular organelle present in most eukaryotic organisms. Since it evolved through the endosymbiosis of bacteria with eukaryotic cells, the mitochondrion has maintained the typical prokaryotic structure, such as a double-membrane structure and a circular double-strand genome [14,15]. The mitochondrial genome (mtDNA) consists of approximately 16.6 kb and encodes 13 essential polypeptides of the oxidative phosphorylation (OXPHOS) system and the necessary RNA machinery [16]. Since mitochondria are considered a cell’s power plant, producing chemical energy in the form of adenine triphosphate (ATP) by OXPHOS, their number within the cells is proportional to the amount of energy required for cellular activities [17,18]. Sperm cells contain, in their midpiece, numerous mitochondria, about 50–75 mitochondria, with, on average, a copy of mtDNA per mitochondrion [19,20,21,22,23]. The main role of sperm mitochondria is to generate the energy for flagellar beating by electron transport chain activity, producing reactive oxygen species (ROS) as a by-product.

Since mtDNA is more prone to the accumulation of mutations than nuclear DNA, an excessive generation of ROS due to sperm mitochondrial dysfunction might damage mtDNA [24,25]. 

Based on this evidence, quantitative and qualitative defects in mtDNA, such as increased mtDNA copies (mtDNAcn), deletions, or point mutations, might severely compromise mtDNA integrity, affecting sperm motility and, in turn, male fertility.

Associations between high mtDNAcn and semen quality have been recently reported. In particular, it has been observed that males with sperm anomalies, mainly asthenozoospermia, showed an increased number of mtDNAcn, and interestingly, the amount of semen defects increased in proportion to the number of mtDNA copies [26,27,28]. Interestingly, although different techniques were used to assess mtDNAcn and the interpretation of semen parameters was based on different WHO guidelines (1999–2021), most of the findings on this topic were similar, hence reporting a negative correlation between mtDNAcn and sperm motility [29,30,31,32,33,34].

Furthermore, alterations in mtDNA such as large deletions, particularly the common deletion of 4977 base pairs (bp), and deletions of 7345 (bp) and 7599 (bp), as well as point mutations, have indeed been highlighted in individuals with reduced sperm motility [35,36,37,38,39,40,41,42].

The cited large-scale deletions encompass, in addition to tRNA genes, genes encoding the respiratory chain. In particular, the common 4977 bp deletion causes the removal or truncation of the genes encoding ND3, ND4L, ND4, COXIII, ATPase 6, and ATP8 proteins. At the same time, 7345 and 7599 bp deletions lead to the loss or truncation of a large part of the proteins composing complex I, complex II, complex III and complex V (ND3, ND4L, ND4, ND5, ND6, Cytb, COXIII, ATPase 6 and 8). Therefore, these three large-scale rearrangements could result in multiple respiratory chain defects [43].

In addition to large deletions, point mutations in genes encoding proteins of OXPHOS have been found in asthenozoospermic males, with most of these mapped mainly in *Cytochrome c oxidase* (*COX*) and *ATP synthase* (*ATP*) genes [11,41,44,45,46,47,48,49]. Although most reported mutations in asthenozoospermic subjects have a very low frequency in the human mitochondrial genome database (MITOMAP), they are mainly classified as benign by predictor protein tools. Therefore, their clinical significance should be further elucidated.

Additionally, the altered expression of TFAM, a nuclear transcription factor that is expressed up to the early spermatid stage and regulates the number of mtDNA transcripts, has been highlighted in subjects with altered copies of mtDNA [33]. In particular, a negative correlation between *TFAM* gene expression and the millions of motile sperm per ejaculate was found.

In conclusion, although anomalies in mtDNA and mitochondrial proteins could affect sperm quality, the inclusion of tests analysing mitochondrial defects in diagnostic routines is still to be evaluated. Although it is well known that mtDNA mutations cause severe clinical manifestations, the association between reduced sperm quality and benign mtDNA variants has been rarely investigated. Therefore, further studies are necessary to identify which genes are more frequently altered in individuals showing reduced sperm quality as the main phenotype. 

Additionally, although qPCR has been considered the gold standard for measuring mtDNAcn, contrary to digital PCR, it does not quantify the absolute copy number of mtDNA. Therefore, it still needs to be better elucidated which method should be introduced as a diagnostic test for measuring mtDNAcn. 

Table 1 summarises the main findings regarding the associations between mitochondrial defects and sperm quality.

### 2.3. Ion Channels

The regulation of ion balance is essential for sperm motility and, thus, correct fertility. In particular, ions pass through channels at least 1000 times faster than through transporters [56]. In mammals, flagellar Ca^2+^ entry is facilitated by the cation channel of sperm (CATSPER), the sperm-specific Ca^2+^ channel complex. 

CATSPER subtypes 1–4 are multi-protein channels codified by different genes, expressed specifically in the testis and located at the plasma membrane of the principal piece in human and mouse sperm flagella, where they are required for sperm hyperactivation [57]. CATSPER was the first ion channel whose gene mutation was associated with reduced sperm motility [58], probably caused by a disruption of the progesterone-sensitive calcium current or the absence of the Catsperβ subunit [59,60]. In particular, the CATSPER subtypes involved in MFI might be CATSPER 1 [61], CATSPER 2 [59], and CATSPER*ε* [62].

SLC26 family members are transporters of small anions, which display wide tissue distribution. As both CFTR and SLC26A3 are expressed in the epithelial cells of the male reproductive tract and in the sperm cells, their respective roles in sperm versus other fertility-related processes are difficult to determine [56]. Mutations in *SLC26* genes, in particular in *SLC26A3*, *SLC9C1*, and *SLC26A8*, are associated with several autosomal recessive disorders, including MFI, due to severe reduced sperm motility [57,63,64,65]. Moreover, mutations in *SLC26A3* can result in congenital chloride diarrhoea associated with subfertility and oligoasthenoteratozoospermia [56]. 

VDACs (voltage-dependent anion-selective channels) are known to mediate anion fluxes in the open state and cations in the closed state. Given the evidence that VDACs can mediate transmembrane Ca^2+^ fluxes [66], they have been intensively studied in mammalian spermatozoa. So far, a few *VDAC* mutations have been associated with reduced sperm motility, particularly alterations of *VDAC2* [67] and *VDAC3* [68].

The last two genes associated with reduced sperm motility are *SLO3* and *PKD*. SLO3, also known as KCNU1, is expressed in the testis and acts as a pH-sensitive channel activated by intracellular alkalisation [69]. A missense variant of *SLO3* was identified in a man with asthenoteratozoospermia [70], probably due to impaired acrosome formation, mitochondrial dysfunction, altered membrane potential during capacitation, and reduced sperm motility [57]. Regarding *PKD*, these genes are mainly expressed in renal epithelial cells, primary cilia and sperm flagella [57]. The members of this protein family, namely PKD1 and PKD2, form a heteromeric calcium channel. Several *PKD1* and *PKD2* mutations have been reported in patients with autosomal dominant polycystic kidney disease and abnormal sperm motility [57].

### 2.4. Proteins of the Flagellum

Mutations of genes encoding proteins of the flagella are mostly involved in the development of MMAF—a condition in which sperm immotility is associated with morphological alterations of the flagella, i.e., primary ciliary dyskinesia (PCD)—since many proteins of the sperm flagella are ciliary components of other cells (in particular, bronchial cells) and DSF—dysplasia of the fibrous sheath. 

#### 2.4.1. MMAF

As introduced above, MMAF is a specific kind of reduced sperm motility characterised by a combination of aberrant flagellar phenotypes (absent, short, angulated, and irregular flagella) that might be easily evidenced during semen routine analysis by optical microscopy and is considered to be a disorder of genetic origin [10]. Currently, more than 30 disease-causing genes have been associated with MMAF (without any other symptoms of PCD) [71,72]. Furthermore, MMAF-associated genes also comprise about half of all confident genes involved in spermiogenesis [73].

Mutations in outer dynein arms and inner dynein arms, responsible for maintaining flagellum/ciliary beat frequency and typical waveform, are genes whose mutation have been mainly reported to result in MMAF. Those genes are *DNAH1*, *DNAH2*, *DNAH6*, *DNAH8*, *DNAH9*, *DNAH17*, *CFAP70*, *CFAP43*, and *CFAP44* [74,75,76,77,78,79,80,81]. As mentioned above, inner and outer dynein arms are multiprotein ATPase complexes that constitute motor proteins and are indispensable for flagellar beating. *DNAH1* was the first gene formally identified in humans as causative for MMAF and MFI [74].

In addition, mutations in genes encoding flagellar proteins, such as AKAP3, AKAP4, and FSIP2 [82,83], and centrosome-related proteins, such as CEP135 and DZIP1 [84,85], have been reported to cause MMAF. Furthermore, as expected in light of the role of radial spokes or central pair complexes, even mutations in those genes might be associated with MMAF. Those genes are *CFAP251*, *CFAP91*, *CFAP65*, *CFAP58*, and *SPEF2* [86,87,88,89,90]. Besides the mutations of the genes reported above, MMAF is associated with mutations of other genes involved in the protein degradation of the definition of sperm localization, such as *TTC21A*, *TTC29*, *CFAP69*, *QRICH2*, *AK7*, *WDR19*, *NDUFA13*, *ARMC2*, and *SEPTIN12* [73,90,91,92,93,94,95,96,97]. Finally, in the last few years, novel genes have been associated with MMAF, such as *CFAP61* [86], *ODF2* [98], *DRC1* [99], *CEP78* [100], *DNAH10* [101], *CFAP47* [102] *STK33* [103], *DNHD1* [72], *CFAP57* [104], *LRRC46* [105], *WDR63* [106], *CFAP206* [107], *SPAG6* [108], *IFT74* [109], *DNALI1* [110], *CCDC40*, *RSPH1* [111], and *GALNTL5* [112]. 

According to this evidence, dynein genes represent the most frequent, albeit not the only, genes mutated in MMAF. In particular, mutations in *DNAH1* seem to be responsible for 25% of instances of MMAF [113]. In the past five years, genetic investigations of male infertility due to MMAF resulted in the identification of almost twenty novel genes, presented above, whose mutations account for up to 30 to 60% of the cases, depending on the analysed cohort. If some of these proteins, such as DNAH1, correspond to well-defined components of the core axoneme, in most cases, the exact functions and molecular mechanisms of the ‘MMAF proteins’ are so far unknown, which constitutes a precious basis for deciphering some of the mechanisms required for flagellum assembly and function [10]. Finally, regarding reproduction outcomes, the mean fertilisation, pregnancy, and live birth rates for patients with MMAF are 63%, 57%, and 43%, respectively [113].

#### 2.4.2. PCD

PCD is an autosomal recessive disorder in which the microtubules of ciliated cells and spermatozoa are immotile with normal morphology and viability, determining normal semen volume and normal sperm concentration but 100% sperm immotility. The reported frequency of PCD in the general population varies between 1 in 10,000 and 20,000 live-born children [114]. Definitive diagnosis must be confirmed using transmission electron microscopy, demonstrating the absence of the dynein arms [115]. The presence of reduced sperm motility might be combined with situs inversus in about half of the cases (so-called Kartagener’s syndrome) and with dysfunction of tracheobronchial cilia, resulting in recurrent episodes of bronchitis and sinusitis, with situs anomalies and congenital cardiac defects, and with recurrent acute, chronic otitis media and some degree of hearing loss [114]. Recently, several genes—necessary for adequate axonemal molecular structure and assembly—have been identified as important, when mutated, in the development of PCD [73,114,116]. The literature reports that about 50% of male patients with PCD are infertile due to a lack of sperm motility [71]. The most common ultrastructural defects of PCD in spermatozoa are a reduction and/or absence of the outer dynein arm (38.5%), reduction and/or absence of both dynein arms (10.5%), microtubule disorganization due to an absence of the inner dynein arm and defects in the central apparatus (14%), absence or interruption of central apparatus (7%), or other rare alterations, such as a reduction and/or absence of the inner dynein arm or oligocilia [114,116,117]. In light of the complexity of the structures involved in sperm motility, it is clear that several genes (about 40) are implicated and have, therefore, been reported to be involved in PCD. The most frequent mutations have been reported in the following genes, which cause PCD in conditions of homozygosis or combined heterozygosis: *DNAH5*, *DNAH11*, *CCDC39*, *DNAI1*, *CCDC40*, *CCDC103*, *SPAG1*, *ZMYND10*, *ARMC4*, *CCDC151*, *DNAI2*, *RSPH1*, *RSPH3*, *CCDC114*, *RSPH4A*, *DNAAF1*, *DNAAF2*, *DNAAF3*, *DNAAF4*, *DNAAF5*, *DNAAF6*, *TTC12*, *DNAJB13*, and *LRRC6* [71,73,114,116]. Taken altogether, *DNAI1* and *DNAH5* abnormalities account for about 30% of PCD cases; however, mutations in the other thirty genes might lead to various ciliary ultrastructural defects and might explain 70% of the genetic causes of PCD [118]. As known and well-evident from our data collection, MMAF pathogenic genes are also often reported to result in PCD or PCD-like symptoms [71,111]. Those genes include genes encoding the axonemal ruler proteins CCDC39 and CCDC30 or genes encoding the proteins SPEF2 [111]. Therefore, dividing genes causing only MMAF and genes causing PCD is not always easy and immediate, and it is possible to define MMAF and PCD as a “phenotypic continuum” [113]. The reported pregnancies in patients with PCD are mostly uneventful, and the children are generally healthy. Notwithstanding, patients affected by PCD are at higher risk of transmitting other defects (such as abnormally positioned internal organs and chronic respiratory tract infections) than infertility, so genetic counselling should be obligatory for couples pursuing assisted reproductive techniques who are affected by this disease [113].

#### 2.4.3. DFS

The fibrous sheath is a peculiar cytoskeletal structure which surrounds the axoneme and outer dense fibres of the sperm flagellum. It underlies the plasma membrane, serving as a scaffold for both glycolytic enzymes and constituents of signalling cascades and having a role in the regulation of sperm motility [118,119]. Dysplasia of fibrous sheath (DFS) is a defect of spermatozoa that was described as a “short tail” or “stump” defect of the sperm flagella [120]. This anomaly is characterised by MFI due to severely reduced sperm motility and often MMAF [121]. Considering the heterogeneity of the described phenotypes, this subtype of reduced sperm motility is often considered part of the MMAF. Few data exist in the literature reporting possible genetic alterations of *AKAP3*, *AKAP4*, *DNAH1*, and *GAPDS* genes [121,122]. Other DFS proteins, such as Sp17 or CABYR or TAKAP-80, have not been associated with reduced sperm motility yet. 

Nevertheless, in light of the frequent classification of DFS among MMAF, we will not discuss those alterations further, referring to DFS as a form of MMAF [10]. DFS has a poor reproductive prognosis, but the number of cases described in the literature is too limited to draw final conclusions [123]. 

#### 2.4.4. Other Genes

Other genes were reported to cause reduced sperm motility without peculiar aspects of MMAF, PCD, or DFS. Those genes, mostly reported in case reports or small cohorts of patients, are *ARL2BP*—which codifies for a ciliary protein and might be involved in the development of syndromic ciliopathy [124], *CTE1/DRC5*, encoding a component of a multiprotein complex which regulates the beating of cilia and flagellum and whose mutation is associated with reduced sperm motility [125], *ADGB*, causing reduced sperm motility by the binding of ADGB to calmodulin [126], and *DNALI1,* whose mutation causes isolated asthenoozospermia due to injury to inner dynein arms [110]. 

In conclusion, in Table 2, we report genetic alterations involved in the development of reduced sperm motility, distinguishing between the well-studied genetic alterations (which are recommended in clinical practice) and the poorly studied genetic alterations identified in single patients or in only few subjects as of yet. To summarise the recommendations regarding genetic analysis that might be used in clinical practice, we referred to clinical guidelines, genetic reviews, and current opinions, besides the opinion of the authors of this manuscript [5,73,113,127].

## 3. Sperm Morphology and Genetics of Reduced Sperm Typical Form

### 3.1. Sperm Morphology

The sperm cell is morphologically divided into two parts: the head and the flagellum. We will briefly delve into the structure of the head of the spermatozoa insofar as it is the part which is mostly abnormal in cases of reduced or abnormal sperm typical morphology.

The sperm head is composed of the plasma membrane (which surrounds all sperm cells), acrosomal vesicle, nucleus, and post-acrosomal region (which connects the head to the flagellum) [9]. The head of mature spermatozoa is the result of spermiogenesis, a complex differentiation process during which the larger, round, and euchromatic head of an early spermatid evolves into the smaller, oval, and heterochromatic head of mature sperm [128]. The morphological classification and study of human sperm heads is very challenging for multiple reasons, such as the size. In fact, the length and width of the sperm head are about 4 µm and 3 µm, respectively [129], therefore representing a non-neglectable issue in the study of the sperm head.

Head integrity is crucial for normal sperm function, and head alterations might lead to MFI. Abnormalities of the sperm head are among the most serious and peculiar alterations of sperm morphology, mostly caused by single-gene defects. Indeed, a better understanding of the physiopathology of head alterations is a prerequisite for improving patient management and genetic counselling and should provide a basis for the development of therapeutic solutions tailored to individual defects [130].

### 3.2. Teratozoospermia

Teratozoospermia is defined as the presence, at semen analysis, of a reduced typical form of spermatozoa. As seen above, this term should not be used anymore. In fact, it should be remembered that the reported “limits” of the semen analysis were not specific [7]. Moreover, the Teratozoospermia Index (TZI) might give additional information to the reporting of the proportion of typical spermatozoa because it is based on the proportions of abnormalities in all regions of the abnormal spermatozoa [8]. Again, even regarding TZI, there is no specific clinical limit to distinguish between normal and pathological results.

From a clinical and practical perspective, alterations of sperm morphology might be subdivided into two categories: polymorphic or monomorphic. Furthermore, there are different subtypes of monomorphic alterations [131], which have, indeed, a strong genetic basis which will be further discussed in detail. It is to be noted that genetic analysis (and, thus, a clinical meaning of the genetic evaluation) should be considered only in patients with 100% (0% typical form of spermatozoa) monomorphic alterations, with some exceptions, which will be discussed above. The following phenotypes might be considered pure monomorphic forms of reduced typical form of spermatozoa: macrozoospermia, globozoospermia, and acephalic spermatozoa [132]. In addition, we introduced the category of “other forms” for sperm morphology alterations, which cannot be associated with any of the three main phenotypes. 

#### 3.2.1. Macrozoospermia

Macrozoospermia is characterised by the presence in the ejaculate of abnormal spermatozoa with an oversized irregular head, abnormal midpiece and acrosome, and multiple flagella [132,133]. This alteration is often associated with oligozoospermia and is a rare clinical condition, affecting less than 1% of patients with MFI [131,134].

The *AURKC* gene, expressed in male germ cells and involved in chromosomal segregation and cytokinesis, was the first recognised genetic alteration in men with macrozoospermia (deletion c.144delC, recently renamed c.145delC) [135]. It is located at 19q13.43 and codifies for aurora kinase C, a member of the aurora kinase family involved in chromosome/chromatid segregation [136]. In particular, AURKC is a component of the chromosomal passenger complex, which has essential functions at the centromere in ensuring correct chromosome alignment and segregation and is required for chromatin-induced microtubule stabilisation and spindle assembly. Moreover, it plays a role in meiosis and in spermatogenesis [131]. Currently, the c.145delC deletion accounts for about 85% of the mutated alleles [132]. Other mutations in men with macrozoospermia have been identified in *AURKC* gene, such as p.C229Y [137], p.Y248 [138], and c436-2A>G [139]. Overall, a positive *AURKC* mutation is found in 50.8–100% of macrozoospermic patients and nearly all positive mutated *AURKC* patients have a typical phenotype, with high percentages of large-head spermatozoa, indicating *AURKC* mutation as the only well-known and well-studied causative genetic mutation of macrozoospermia [5,132]. Mutations in *AURKC* genes are mainly located (up to 97%) in exons 3 and 6 [133]. 

Interestingly, a recent study analysing 599 infertile men reported that *AURKC* mutations are more frequent than Klinefelter syndrome and constitute the leading genetic cause of MFI in North Africa [140], albeit, in this study, karyotyping and Y-microdeletion diagnoses were not studied in all patients. This high frequency might be due to a selective advantage for heterozygous carriers of *AURKC* mutations. 

In addition to *AURKC*, other genes, such as *ZMYND15*, *NUP210L*, *MEIKIN*, *ADAD2*, and *MDC1*, might be involved in macrozoospermia, albeit mutations in these genes have been identified in a few case reports [133,141].

The presence of at least 70% of spermatozoa with a large head is usually associated with *AURKC* mutations [134,142]. According to the most recent literature regarding macrozoopsermia [133], genetic analysis (Sanger sequencing of exons 3 and 6) should be performed in patients with more than 20% macrocephalic and more than 10% multiflagellata spermatozoa, followed by exome sequencing analysis in the case of a negative result. 

The clinical follow-up of patients with macrozoospermia is often associated with negative reproductive outcomes, and, in a recent literature review, very few pregnancies resulted in live births [134]. In fact, spermatozoa from patients with homozygous c.145delC variants are usually tetraploid, making assisted reproductive techniques unsuccessful in these patients [133]. 

#### 3.2.2. Globoozoospermia

First described in humans in 1971, globozoospermia is a very rare condition, observed in less than 0.1% of cases of MFI. It is characterised by round-headed spermatozoa with an absent acrosome, an aberrant nuclear membrane and midpiece defects [5,113,131]. 

Globozoospermia has been catalogued as type I when round-headed spermatozoa lack the acrosome and acrosomal enzymes and type II when they have some remnants of the acrosome [143]. Another classification distinguishes between classic form (100% round-headed spermatozoa, which are unable to penetrate the oocyte) and partial form (20–90% of morphologically abnormal spermatozoa) [144]. In clinical practice, globozoospermia is diagnosed by semen analysis when a minimum of 20–50% of round-headed sperm cells is found in the ejaculate [130]. 

The sperm without acrosome is unable to go through the zona pellucida, often inducing fertilisation failures even when ICSI is attempted. In particular, the mean fertilisation rate for patients with total globozoospermia is 24.1%, whilst the fertilisation rate is higher in patients with partial globozoospermia (61%) [145]. The association of ICSI with assisted oocyte activation significantly improved the mean fertilisation rate (58.8% ± 23.7%) [144,145].

To our knowledge, several genes, reported here below, have been identified to be associated with globozoospermia in humans so far [146]. 

*SPATA16* was first identified in 2007 as being mutated in three brothers affected with globozoospermia [147]. In 2010, Liu et al. [148] reported a mutation in *PICK1* in one patient with globozoospermia. These two genes seem to be involved in the same process of spermiogenesis [131]. Furthermore, in 2011, another gene, *DPY19L2*, was reported to be deleted in four globozoospermic brothers and in three additional unrelated patients [149]. The mechanism underlying this deletion is due to non-allelic homologous recombination between two low copy repeats that share 96.5% identity and flank the *DPY19L2* locus [149]. Currently, both recessive deletions and mutations in *DPY19L2* seem to be the main cause of globozoospermia. DPY19L2 is a transmembrane protein expressed in the testis [150], which is involved in the anchorage of the cytoskeleton to the nuclear membrane. Therefore, its absence/mutation leads to instability and the dissociation of the layered structure of acroplaxome, further resulting in the formation of round head spermatozoa [151]. A wide spectrum of plausible mutations of *DPY19L2* has been detected in globozoospermic individuals: deletion of the whole locus, nonsense, missense, splicing mutations, and partial deletion encompassing exons 8, 9, 11, 15, 21, and intron 11 [150]. In addition, Li and colleagues reported the case of a patient with globozoospermia presenting a compound heterozygous mutation of the *DNAH6* gene [76] and suggesting *DNAH6* as a novel candidate gene for globozoospermia. Recently, Oud studied the mutations of different genes in 15 patients with globozoospermia and found mutations in 7 genes, besides *SPATA16*, *DPY19L2*, *PICK1*, and *DNAH6*, considered as possible candidates in the pathogenesis of globozoospermia; those genes are *ZPBP*, *CCDC62*, *C2CD6*, *CCIN*, *C7orf61*, *DNAH17*, and *GGN* [146,152]. They might, indeed, have a role in the development of globozoospermia [152]. Other putative genes that have usually been described in case reports or in small series of patients are *CCNB3*, *PIWIL4*, *CHPT1* [152], *SSFA2* [153], *SPACA1* [154], *SPATA 20* [155], and *FSIP2* [80]. 

Notwithstanding the evidence reported above, as previously exposed, the gene most frequently involved in the pathogenesis of globozoospermia is *DPY19L2* [5,113,143,145]. In particular, it is mutated in about 80% of patients with globozoospermia type I [143], and the mutation most frequently described is the deletion of the entire gene, which has been reported to have a prevalence ranging from 22.2% [156,157]. The discrepancy might be due to the difficulty in the identification of the percentage of patients with total or partial form globozoospermia among the studies and the geographical origins or the studied cohorts of patients. In conclusion, according to the evidence reported so far, besides *DPY19L2*, other genes that might be confidently associated with globozoospermia in humans, and whose analysis might be recommended in clinical practice, are *SPATA16*, *PICK1*, *ZPBP1*, and *CCDC62* [130,158]. This might be expected, in the light of the evidence that those genes seem to have interconnected functions. For instance, Z*PBP* forms part of the same gene interaction network with *SPATA16* and *DPY19L2*, thus suggesting they might be mechanistically associated [146]. 

#### 3.2.3. Acephalic Spermatozoa

Acephalic spermatozoa syndrome (ASS) is often considered to be the most severe form of the reduced and abnormal typical form of spermatozoa and is defined as the presence of the predominance of headless spermatozoa in the ejaculate [130,159]. This type of abnormal sperm morphology was first described as “pin head spermatozoa” and later renamed “decapitated spermatozoa” or acephalic spermatozoa syndrome [159].

The essence of ASS is the abnormal development of the sperm head–tail coupling apparatus. Moreover, ASS is classified into three subtypes (I, II, and III) according to the broken points in said apparatus [159].

The genetic origin of ASS was first identified in humans in 2016, showing that biallelic *SUN5* mutations might be responsible for ASS [160]. According to the reported cases so far, approximately 40–50% of human ASS are caused by SUN5 mutations/deletions [159,161]. *SUN5* is a gene encoding for a transmembrane protein expressed in the testis, localised in the head–flagellum junction of the sperm [161], which might take part in the attachment of the sperm flagellum to the head and might be involved in the assembly of the sperm neck and in the nuclear envelope reconstitution and nuclear migration [159,162]. In *SUN5*-knockout mice or in patients with pathogenic *SUN5* mutations, it was observed that the sperm head separated from the tail because the flagellum could not attach to the nuclear envelope [160,163].

The other strongly validated pathogenic genes of ASS are *PMFBP1* and *TSGA10.* PMFBP1, first reported in 2018 [164], is a testis-specific protein localised at the head–tail connecting piece of sperm, and its mutations are responsible for approximately 35% of reported cases of human ASS [160] whilst *TSGA10* [165] is a gene encoding a protein localised in the principal piece to the midpiece of the sperm. 

Other genes were reported to be mutated singles in patients with ASS, such as *BRDT* [166], *HOOK1* [167], *CEP112* [100], *ACTRT1* [168], *SPATC1L* [169], and *SPATA20*; they were even associated with partial globozoospermia [170]. Furthermore, though the knockout mice of several genes were reported to have a phenotype analogous to ASS, most of their mutations were not found to be associated with ASS in humans [159]. To date, because of defects in both sperm structures and functions, no spontaneous pregnancy has been reported in a couple wherein the husband had ASS [159]. Nevertheless, satisfactory outcomes of clinical pregnancy were reported in patients with ASS using ICSI [171], albeit the results of the ICSI in patients with ASS were conflicting due to both positive (in particular ASS subtype II) and negative (in particular AAS subtype I and III) reports on the ICSI outcomes [159,161].

#### 3.2.4. Other Forms of Reduced Typical Sperm Morphology 

Besides macrozoospermia, globozoospermia, and ASS, a limited number of studies have focused on other morphology alterations, identifying other genes that might be involved in the development of other forms of abnormal sperm morphology. These genes, reported only in case reports or in small cohorts of patients, are *BSCL2*, identified in abnormal sperm heads with absent hooks, banana shapes, or apparent cavities [172]; *SEPTIN 14*, whose mutations lead to anomalies in sperm head morphology [173]; *FBOX43*, involved in amorphous-headed spermatozoa [174]; *AMZ2,* involved in vacuolated spermatozoa [175]; *RNF220*, whose loss-of-function mutation might induce the formation of small-headed sperm [176]; *CALICIN*, involved in the development of severe head malformation [177]; WRD12, associated with tapered-head spermatozoa [178]; *AGTPBP1*, associated with sperm head and flagella defects [179]; and *ACTL7A*, involved in the development of bubble-shaped acrosomes [180]. 

Finally, needle spermatozoa are characterised by small head deformities, tapered heads, and irregular shapes. Whilst in animal models, *SPATA6* deletion has been associated with the development of needle spermatozoa, no human data regarding needle spermatozoa are available yet [127].

In conclusion, in Table 3, we report genetic alterations involved in the development of abnormal sperm morphology, distinguishing between the well-studied genetic alterations (which are, indeed, recommended in clinical practice) and the poorly studied genetic alterations identified in single patients or few subjects as of yet. To summarise the recommendations regarding genetic analysis that might be used in clinical practice, we referred to clinical guidelines, genetic reviews, and current opinions, besides the opinion of the authors of this manuscript [13,73,113,127,143].

## 4. Final Considerations

Reduced sperm motility and reduced typical sperm morphology, as seen above, are rarely caused by genetic alterations in male infertile patients. Genetic analysis in the context of MFI should always be thought as III-level analysis, after the first clinical approach to the patient with MFI and I-level tests (accurate anamnesis, physical examination, complete semen analysis, semen culture with atypical pathogens and HPV, scrotal doppler ultrasound, transrectal prostatic ultrasound) and II-level tests (analysis of genetics aneuploidies and evaluation of oxidative stress). The correct diagnostic picture is, therefore, a multi-step process, in which the genetic analysis dwells after the correct identification of subjects to be tested and the right application of genetic tests based on clear clinical data [5].

**Figure 2:**
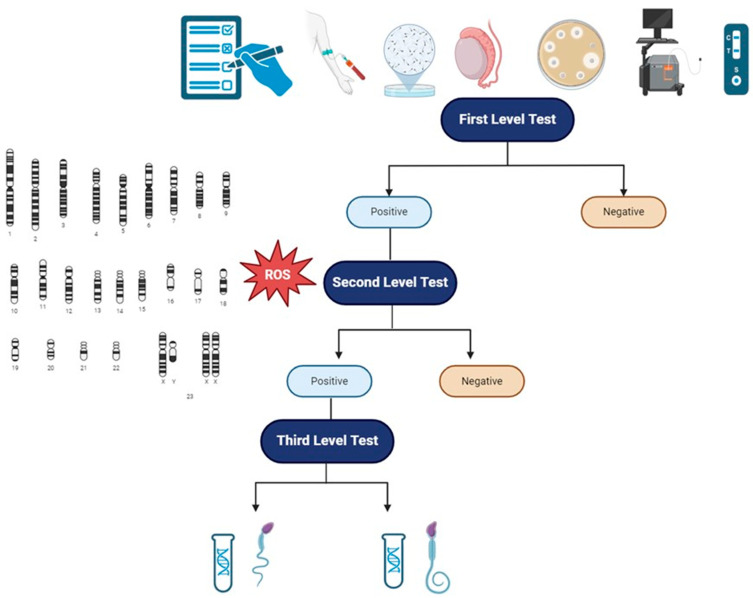
Clinical and diagnostic flowchart to patients with MFI, in particular to patients with isolated/reduced sperm motility or reduced typical sperm morphology. I-level tests: anamnesis, physical examination, scrotal and prostatic ultrasound evaluation, semen analysis, semen microbiological evaluation with HPV. II-level tests: evaluation of oxidative stress and spermatic aneuploidy. III-level tests: genetic analysis. Abbreviations: ROS—reactive oxygen species.

In light of this current opinion and recommendation, we propose (Figure 2) a practical flowchart in order to approach patients with reduced sperm motility or reduced typical sperm morphology. We further highlight the fact that those genetic analyses have to be requested in specific patients as a III-level exam. Moreover, we underline that the approach to patients with MFI must be considered a couple-oriented approach, evaluating both the male and the female factors so as to consider the infertile couple as a single entity. Finally, besides the possible definition of a correct diagnosis and correct management of the patients, a genetic analysis might be useful to define the early management of potential comorbidities; assess congenital, reproductive, and general health risks to the future offspring; and offer genetic testing of family members [181]. 

## 5. Conclusions

The genetic contribution to MFI is noteworthy, with several genes that might be analysed in clinical practice, albeit genetic analyses currently recommended in clinical practice are still few. Isolated reduced sperm motility and isolated reduced typical sperm morphology are semen alterations with a strong genetic basis. Notwithstanding, the approach to patients with MFI has to be a couple-oriented approach, and genetic analysis has to be considered as a III-level test after the correct identification of subjects to be tested.

Further well-designed studies on patients with different semen alterations categorized into defined subtypes of male infertility should be performed to gain evidence for future implementation in the diagnostic routine of gene panel assays.

## Figures and Tables

**Table 1:** Alterations in mtDNA associated with reduced sperm motility.

References	Mitochondrial Defects	Main Findings	Suggestions
[29,30,31,32,33,34,50,51,52]	Altered mtDNA content	High number of mtDNA copies in men with reduced sperm motility	Not recommended yet
[35,37,39,40,41,42,53,54,55]	Large mtDNA deletions or single mutations in genes encoding mitochondrial proteins	Associations with reduced sperm motility	Not recommended yet

**Table 2:** Mechanisms and genes involved reduce sperm motility. Abbreviations: MMAF: multiple morphological alterations of flagella; mtDNA: mitochondrial DNA; PCD; primary ciliary dyskinesia.

Mechanism Involved	Genes Involved	Currently Used in Clinical Practice
Ion channels	*CATSPER 1-2-ε*, *SLC26A3*, *SLC9C1*, *SLC26A8*, *VDAC2-3*, *SLO3*, *PKD1-2*	Recommended
Proteins of the flagella	*DNAH5*, *DNAH11*, *CCDC39*, *DNAI1*, *CCDC40*, *CCDC103*, *SPAG1*, *ZMYND10*, *ARMC4*, *CCDC151*, *DNAI2*, *RSPH1*, *RSPH3*, *DNAH1*, *DNAH2*, *DNAH6*, *DNAH8*, *DNAH9*, *DNAH17*, *CFAP70*, *CFAP43*, *CFAP44*, *CCDC114*, *RSPH4A*, *DNAAF1*, *DNAAF2*, *DNAAF3*, *DNAAF4*, *DNAAF5*, *DNAAF6*, *TTC12*, *DNAJB13*, *LRRC6*, *AKAP3*, *AKAP4*, *FSIP2*, *CEP135*, *DZIP1*, *CFAP251*, *CFAP91*, *CFAP65*, *CFAP58*, *SPEF2*, *TTC21A*, *TTC29*, *CFAP69*, *QRICH2*, *AK7*, *WDR19*, *NDUFA13*, *ARMC2*, *SEPTIN12*	Recommended
Other causes	*GAPDS*, *ARL2BP*, *CTE1/DRC5*, *ADGB*, *DNALI1*, *CFAP61*, *DRC1*, *CEP78*, *DNAH10*, *CFAP47*, *STK33*, *DNHD1*, *CFAP57*, *LRRC46*, *WDR63*, *CFAP206*, *SPAG6*, *IFT74*, *DNALI1*, *CCDC40*, *RSPH1*, *GALNTL5*	Not Recommended Yet
**Form of AZS**	**Genes Involved**	**Currently Used in Clinical Practice**
MMAF without PCD	*DNAH1*, *DNAH2*, *DNAH6*, *DNAH8*, *DNAH9*, *DNAH17*, *CFAP70*, *CFAP43*, *CFAP44*, *AKAP3*, *AKAP4*, *FSIP2*, *CEP135*, *DZIP1*, *CFAP251*, *CFAP91*, *CFAP65*, *CFAP58*, *SPEF2*, *ARMC2*, *CFAP69*, *QRICH2*, *SEPTIN12*, *TTC29*	Recommended

**Table 3:** Genetic alterations causing monomorphic forms of abnormal sperm morphology.

Form of Abnormal Sperm Morphology	Genes Involved	Current Analysis in Clinical Practice
Macrozoospermia	*AURKC*	Recommended
	*ZMYND15*, *NUP210L*, *MEIKIN*, *ADAD2*, *MDC1*	Not recommended yet
Globozoospermia	*DPY19L2*, *SPATA16*, *PICK1*, *ZPBP*, *CDC62*	Recommended
	*C2CD6*, *CCIN*, *C7orf61*, *DNAH17*, *GGN*, *CCNB3*, *PIWIL4*, *CHPT1*, *SSFA2*, *SPACA1*, *SPATA20*, *FSIP2*	Not recommended yet
Acephalic spermatozoa syndrome	*SUN5*, *PMFBP1*, *TSGA10*	Recommended
	*BDRT*, *HOOK1*, *CEP112*, *ACTRT1*, *SPATC1L*, *SPATA20*	Not recommended yet
Other forms of abnormal sperm morphology	*BSCL2*, *SEPTIN14*, *FBOX43*, *AMZ2*, *RNF220*, *CALICIN*, *WDR12*, *ACTL7A*, *SPATA6*	Not recommended yet

## Data Availability

Not applicable.
